# Editorial: Insights in microbiotechnology: 2022

**DOI:** 10.3389/fmicb.2024.1293087

**Published:** 2024-05-29

**Authors:** Muhammad Bilal

**Affiliations:** ^1^Department of Sanitary Engineering, Faculty of Civil and Environmental Engineering, Gdansk University of Technology, Gdańsk, Poland; ^2^EkoTech Center, Gdańsk University of Technology, Gdańsk, Poland

**Keywords:** glycoside hydrolases, microbial communities, environmental microbiology, omic technologies, gut microbiome, multi-omics, cyanobacteria, bioremediation

Microbiotechnology is the implementation of biological and technological principles to manipulate microorganisms, such as bacteria, fungi, viruses, and algae, for various industrial and environmental applications. In recent decades, microbiotechnology has experienced a paradigm shift toward synthetic biology, precision microbiome engineering, personalized medicine, and sustainable bioprocessing techniques with the advent of rapid and extensive development of state-of-the-art microbial and OMICs approaches. OMICs technologies are becoming more prevalent in all facets of microbiotechnology, contributing meaningful datasets in accelerating our understanding and excel in the subject. These technologies are used either DNA or RNA based methods like metagenomics or metatranscriptomics, respectively. However, with the introduction of high-throughput techniques in other disciplines, OMICs technologies now encompass metabolomics, metaproteomics, phenomics, epigenetics, and other emerging omics studies (Altermann and Hickey, 2020). Using these novel technologies, abundance of microorganisms have been thoroughly studied and used in a wide variety of applications, such as agricultural productivity, plant protection, pollution remediation, and the improvement of plant and human health (Afridi et al., 2022; Anand et al., 2022; Hadibarata et al., 2022; Poppeliers et al., 2023; Rafeeq et al., 2023).

By issuing this call for papers on “*Insights in Microbiotechnology-2022*” in the esteemed journal, Frontiers in Microbiology, we sought contributions in the form of research, reviews, mini-reviews, perspectives, and opinions to provide a comprehensive overview of the current state and prospects of the field. This subject explicitly encompasses the most noteworthy scientific discoveries, breakthroughs, challenges, advancements, and potential avenues within the realm of microbiotechnology throughout the year 2022.

The carbohydrate-active enzymes known as glycoside hydrolases (GHs) are crucial for a variety of biotechnological and environmental activities, including the production of biofuels and the carbon cycle. Bacteria need numerous enzymes working in a coordinated manner to complete carbohydrate processing. Berlemont conducted a study to investigate the distribution pattern of 406,337 GH genes and their association with transporter genes in 15,640 fully sequenced bacterial genomes. Although the levels of clustered and scattered GH-genes were conserved throughout different bacterial lineages, the overall occurrence of GH-gene clustering was generally higher than that in randomized genomes. The genomic adaptations for carbohydrate processing in bacteria with the most identified GH-genes also demonstrated the diverse environmental origin of the sequenced strains, including soil and mammal gut. Most scattered genomes are linked to soil ecosystems, whereas most clustered genomes come from aquatic environments and animal GIT. Therefore, the microorganisms with the most significant potential for carbohydrates adapted to their environments in various ways.

In order to create synthetic microbial communities (SynCom), De la Vega-Camarillo et al. used a set of plant growth-promoting bacteria, which were isolated from the Jala maize landrace. Three SynCom were constructed based on the phenotypic characteristics of various bacterial strains, including *Achromobacter xylosoxidans* Z2K8, *Burkholderia* sp. Z1AL11, *Kosakonia pseudosacchari* Z2WD1, *Klebsiella variicola* R3J3HD7, *Pantoea* sp. E2AD2, *Pantoea ananatis* E2HD8, *Pseudomonas protegens* E2HL9, *P. protegens* E1BL2, and *Phytobacter diazotrophicus* Z2WL1. In gnotobiotic and greenhouse trials, all SynCom designs increased maize growth. Nonetheless, SynCom 3 created with *A. xylosoxidans* Z2K8, *Burkholderia* sp. Z1AL11, *K. variicola* R3J3HD7, *P. protegens* E1BL2, *P. diazotrophicus* Z2WL1, and *P. ananatis* E2HD8 demonstrated the highest outcomes for stimulating plant growth, yield, and fungal rust suppression. The findings proved that SynCom assemblies containing culturable bacteria from indigenous maize landraces might promote plant growth, fostering a more sustainable and economically viable approach to agriculture.

In recent years, omics technologies have emerged as a powerful tool within the sphere of environmental microbiology to expand our knowledge of exploring the interactions between unculturable organisms and their environment *in vivo*. Abdullah et al. utilized omics technologies to process meta-data, envision critical trends related to cold climate bioremediation, and investigate novel metabolic degradation pathways. Given the crucial role of the gut microbiome in health and disease, it is indispensable to comprehend the factors that influence the gut microbiota and the microenvironment. Qiu et al. applied an integrative multi-omics approach to evaluate the functional and metabolic diversity of microbiomes in the gut microenvironment and scrutinized the relationship between the host and intestinal flora.

Ciani and Adessi illustrated the most recent tendencies and perspectives in the cyanobacteria-assisted bioremediation process of heavy metals and the efficient recovery and reuse of metals. They directed that heavy metal biosorption by cyanobacteria can be integrated with the consecutive valorization of obtained metalorganic materials to achieve value-added compounds, thus prospecting the field of phyconanotechnology. Altogether, all articles published in this Research Topic highlight the significance of microorganisms in a variety of biotechnological and environmental applications.

This Research Topic serves as an invaluable resource for readers interested in staying updated with the latest progress and developments in the field of microbiotechnology. It spotlights the innovative research conducted by up-and-coming experts in the field, specifically emphasizing the transforming abilities of microorganisms that greatly influence the scientific community. The advent of multi-omic technologies has revolutionized microbiotechnology, allowing for a comprehensive understanding of the molecular landscape. Additional research is required to explore artificial intelligence, machine learning, multidimensional statistical techniques, and user-friendly analytical tools for uncovering the correlation between microbial metabolites and the host, as well as gaining a deeper comprehension of microbial interaction with environmental pollutants.

## Author contributions

MB: Conceptualization, Investigation, Writing – original draft, Writing – review & editing.
